# Supplementary material for: Molecular basis for PrimPol recruitment to replication forks by RPA
Source: Nat Commun. 2017 May 23;8:15222. doi: 10.1038/ncomms15222 (PMC5457501; doi:10.1038/ncomms15222)
Supplement: Supplementary Information — Supplementary figures and supplementary table. [file ncomms15222-s1.pdf]

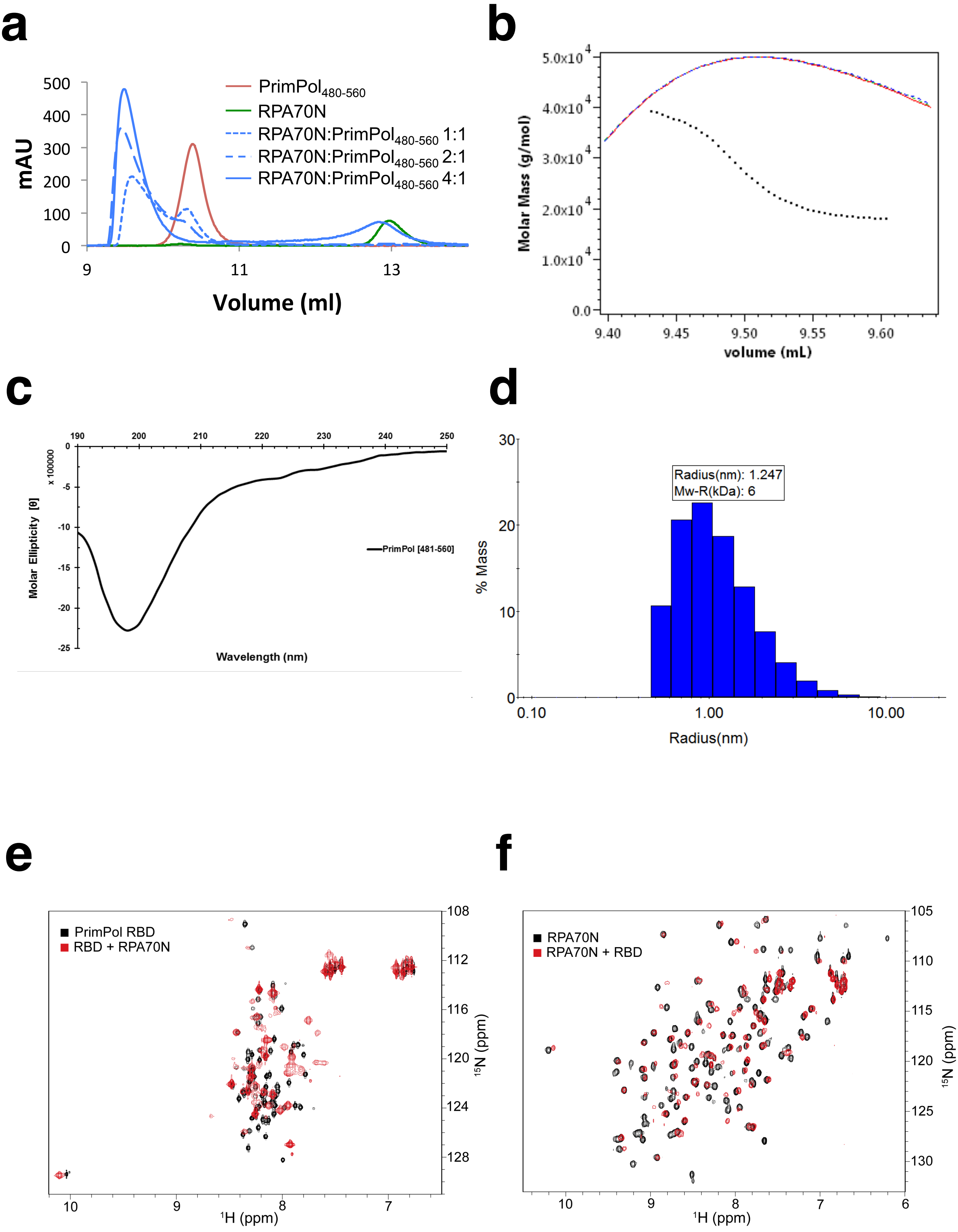
a
500
400
300
200
100
0
mAU
9
11
13
Volume (ml)
PrimPol480-560
RPA70N
RPA70N:PrimPol480-560 1:1
RPA70N:PrimPol480-560 2:1
RPA70N:PrimPol480-560 4:1
b
5.0x10^4
4.0x10^4
3.0x10^4
2.0x10^4
1.0x10^4
0.0
Molar Mass (g/mol)
9.40
9.45
9.50
9.55
9.60
volume (mL)
c
190
200
210
220
230
240
250
x 100000
0
-5
-10
-15
-20
-25
Molar Ellipticity [θ]
PrimPol [481-560]
Wavelength (nm)
d
30
20
10
0
% Mass
Radius(nm): 1.247
Mw-R(kDa): 6
0.10
1.00
10.00
Radius(nm)
e
PrimPol RBD
RBD + RPA70N
108
112
116
120
124
128
15N (ppm)
10
9
8
7
1H (ppm)
f
RPA70N
RPA70N + RBD
105
110
115
120
125
130
15N (ppm)
10
9
8
7
6
1H (ppm)

Supplementary Figure 1. PrimPol's RBD interacts with RPA70N. (**a**) Chromatograph showing the retention volumes of the PrimPol RBD (residues 480-560) (Red), RPA70N (Green) and RBD titrated with varying molar ratios of RPA70N (Blue). (**b**) MALS analysis of the major peak eluted from the saturated RBD-RPA70N E7R sample at a 1:4 molar ratio. The observed Refractive Index (blue), UV (green), and Light Scattering (red) readings were used to calculate the molecular weight of the complex over the course of elution (black). (**c**) Circular dichroism spectrum of 20 μM $PrimPol_{RBD}$ collected between 190-250 nm showing a spectral shape characteristic of an unstructured protein lacking any significant α-helical or β-strand propensity. (**d**) Regularization graph of 10 accumulations of dynamic light scattering data collected on $PrimPol_{RBD}$ samples at 200 μM. A single peak was observed with a radius of 1.25 nm that corresponds to a predicted molecular weight of ~6 kDa when modelled with random coil protein shape algorithms. This is close to the expected 8.8 kDa of the monomeric protein. (**e**) ^{15}N-^{1}H HSQC spectra of ^{15}N-enriched PrimPol RBD in the absence (black) and presence (red) of unlabelled RPA70N (70N) titrated at a 2:1 ratio. (**f**) ^{15}N-^{1}H HSQC spectra of ^{15}N-enriched RPA70N in the absence (black) or presence (red) of 2-fold molar excess of unlabelled PrimPol RBD.

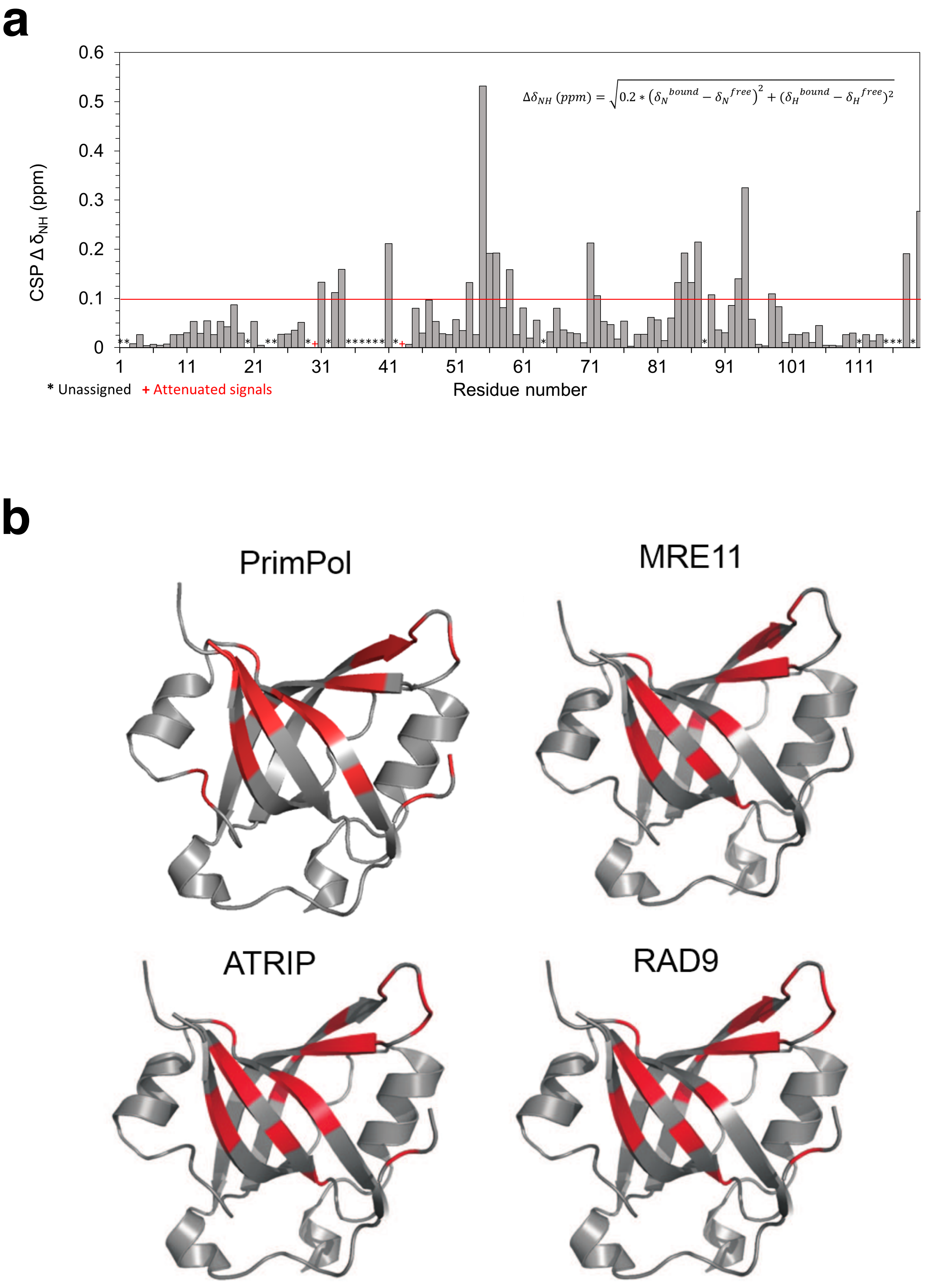

a
0.6
0.5
0.4
0.3
0.2
0.1
0
CSP Δ δNH (ppm)
ΔδNH (ppm) = √(0.2 * (δN^bound − δN^free)^2 + (δH^bound − δH^free)^2)
1
11
21
31
41
51
61
71
81
91
101
111
* Unassigned
+ Attenuated signals
Residue number
b
PrimPol
MRE11
ATRIP
RAD9

Supplementary Figure 2. PrimPol interacts with RPA70N in the same region as other RPA70N binding partners. (**a**) Chemical shift perturbations (CSPs) observed in the ^{15}N-^{1}H HSQC NMR spectra following titration of PrimPol RBM-A peptide quantified and plotted versus RPA70N residue number. (**b**) Structure of RPA70N mapped with CSPs above a $\Delta\delta$ threshold of 0.1 ppm shown in red. The interacting region of PrimPol is similar to that of other binding partners such as ATRIP, RAD9, and MRE11, which bind mostly on one side of the β-barrel.

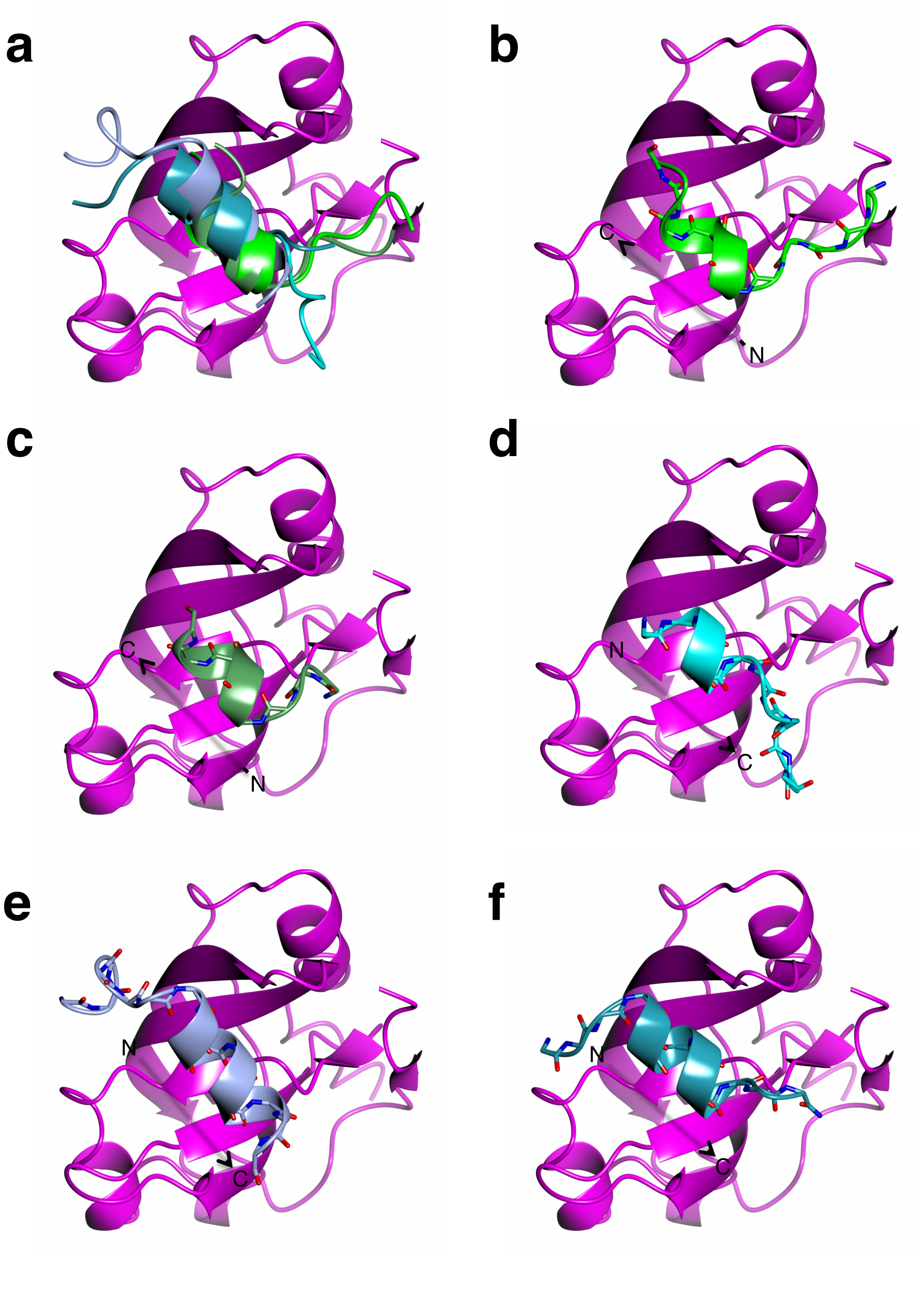

a
b
C
N
c
C
N
d
N
C
e
N
C
f
N
C

Supplementary Figure 3. RBMs bind with a reverse polarity to the basic cleft of RPA70N. Ribbon representations of RPA70N^{E7R} from the current PrimPol$_{514-528}$ peptide complex superposed with helical elements from previously established RPA70N protein complexes. The RBM helices are seen to bind in reverse polarity to these established complexes. The RPA70N is coloured magenta. (**a**) All the helices from the different RPA70N complexes superposed: PrimPol$_{514-528}$ peptide complex coloured light green, PrimPol$_{480-560}$ coloured dark green, Peptide from Dna2 (PDBID: 5EAY) coloured cyan, p53N (fragment 33-60) (PDBID: 2B3G) coloured sky blue, 3,4 dichlorophenyalanine ATRIP derived peptide (PDBID: 4NB3) coloured turquoise. (**b-f**) Combined ribbon and main chain representations of: (**b**) The current PrimPol$_{514-528}$ peptide complex. (**c**) The PrimPol$_{480-560}$ complex. (**d**) RPA70N binding a peptide from Dna2 (PDBID: 5EAY). (**e**) RPA70N binding p53N (fragment 33-60) (PDBID: 2B3G). (**f**) RPA70N binding 3,4 dichlorophenyalanine ATRIP derived peptide (PDBID: 4NB3).

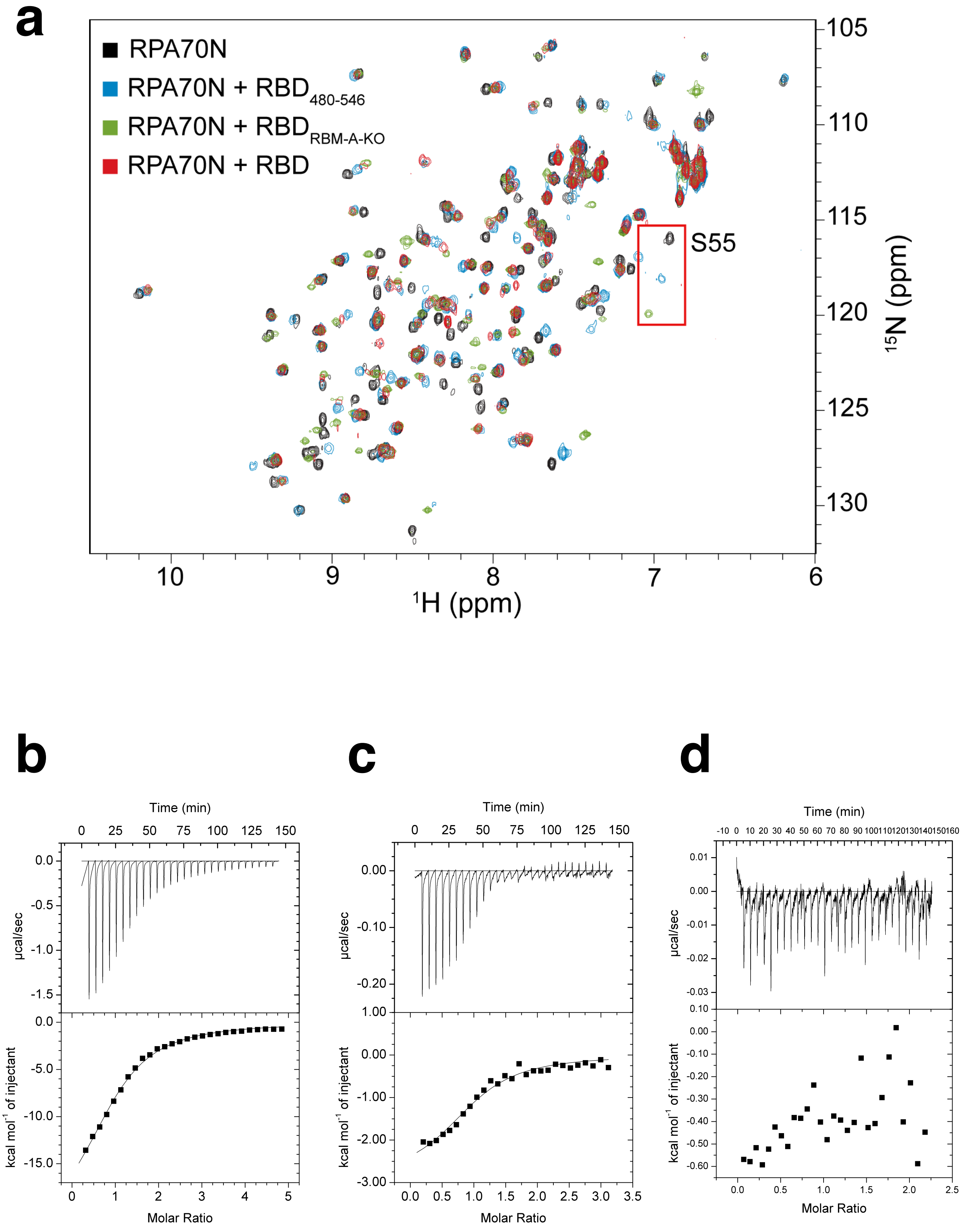

a
RPA70N
RPA70N + RBD480-546
RPA70N + RBDRBM-A-KO
RPA70N + RBD
S55
105
110
115
120
125
130
15N (ppm)
10
9
8
7
6
1H (ppm)
b
Time (min)
0 25 50 75 100 125 150
µcal/sec
0.0
-0.5
-1.0
-1.5
kcal mol-1 of injectant
0.0
-5.0
-10.0
-15.0
0 1 2 3 4 5
Molar Ratio
c
Time (min)
0 25 50 75 100 125 150
µcal/sec
0.00
-0.10
-0.20
kcal mol-1 of injectant
1.00
0.00
-1.00
-2.00
-3.00
0.0 0.5 1.0 1.5 2.0 2.5 3.0 3.5
Molar Ratio
d
Time (min)
-10 0 10 20 30 40 50 60 70 80 90 100110120130140150160
µcal/sec
0.01
0.00
-0.01
-0.02
-0.03
kcal mol-1 of injectant
0.10
0.00
-0.10
-0.20
-0.30
-0.40
-0.50
-0.60
0.0 0.5 1.0 1.5 2.0 2.5
Molar Ratio

Supplementary Figure 4. RPA70N dynamically interacts with both RBM-A and RBM-B. (**a**) ^{15}N-^{1}H HSQC overlay comparing RPA70N bound to wild type RBD or constructs mutated to inhibit binding to RBM-A or B. The RPA70N spectrum (black) shows distinct chemical shift perturbations (CSPs) when titrated with 2-fold molar excess of RBD constructs that select for binding to only RBM-A (blue) or RBM-B (green). These are different from the combination of signal shifting and broadening induced by the wild type RBD (red), which is indicative of dynamic exchange between RBMs. (**b-d**) Isothermal titration calorimetry data showing the heat of binding evolved upon titrating a cell containing 1.4 mL of 20 μM RPA70N with iterative 10 μL injections of 400 μM PrimPolRBD mutants (**b**) $PrimPol_{480-546}$ (B-K.O.), (**c**) RBM-A-K.O. and (**d**) RBM-A/B-K.O. Dissociation constants were calculated with a single site binding model to give statistically equivalent values of 7.8 ± 0.6 *μ*M and 6.7 ± 1.5 *μ*M for the A-K.O. and B-K.O. mutant domains, respectively. The double knockout RBM-A/B-K.O. showed no significant heat evolved upon titration, indicating that no interaction was observed.

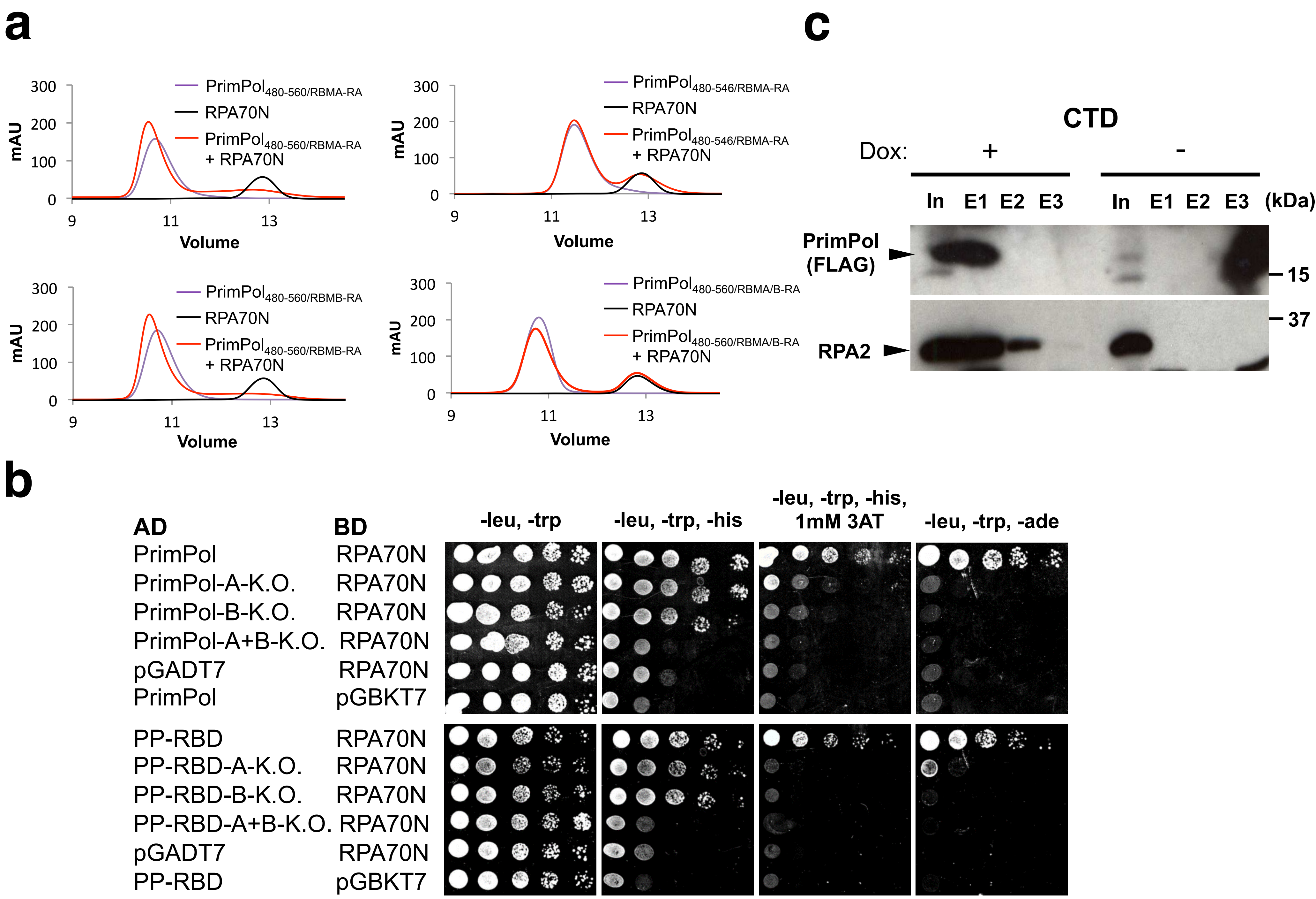
a
PrimPol480-560/RBMA-RA
RPA70N
PrimPol480-560/RBMA-RA + RPA70N
PrimPol480-546/RBMA-RA
PrimPol480-546/RBMA-RA + RPA70N
PrimPol480-560/RBMB-RA
PrimPol480-560/RBMB-RA + RPA70N
PrimPol480-560/RBMA/B-RA
PrimPol480-560/RBMA/B-RA + RPA70N
mAU
Volume
b
AD
BD
-leu, -trp
-leu, -trp, -his
-leu, -trp, -his, 1mM 3AT
-leu, -trp, -ade
PrimPol RPA70N
PrimPol-A-K.O. RPA70N
PrimPol-B-K.O. RPA70N
PrimPol-A+B-K.O. RPA70N
pGADT7 RPA70N
PrimPol pGBKT7
PP-RBD RPA70N
PP-RBD-A-K.O. RPA70N
PP-RBD-B-K.O. RPA70N
PP-RBD-A+B-K.O. RPA70N
pGADT7 RPA70N
PP-RBD pGBKT7
c
CTD
Dox: + -
In E1 E2 E3 In E1 E2 E3 (kDa)
PrimPol (FLAG)
RPA2
15
37

Supplementary Figure 5. PrimPol's RBMs interacts with RPA *in vivo*. (**a**) Validation of RBMA/B-RA mutants used for *in vivo* analysis. Chromatographs showing the retention volumes of $\text{PrimPol}_{480\text{-}560}$ RBMA/B-RA mutants (D519R/F522A and D551R/I554A) in the presence (red) and absence (purple) of RPA70N (black) in a 1:1 ratio. $\text{PrimPol}_{480\text{-}560}$ RBMA-RA (D519R/F522A) (top-left panel) and $\text{PrimPol}_{480\text{-}560}$ RBMB-RA (D551R/I554A) (bottom-left panel) are still able to bind RPA70N when mutated in isolation. However, when the RBMA-RA mutation is introduced on $\text{PrimPol}_{480\text{-}546}$ (top-right panel) or in combination with RBMB-RA (bottom-right panel), binding to RPA70N is lost. These results validate the use of the D519R/F522A and D551R/I554A mutations used for *in vivo* analysis. (**b**) PrimPol interacts with RPA70N in the yeast two-hybrid assay. To study the interaction with RPA70N, either full-length PrimPol (upper panels) or its RBD (PP-RBD – a.a. 480-560; lower panels) were used. The following amino acids of PrimPol were mutated - D519R and F522A in A-K.O.; D551R and I554A in B-K.O.; D519R, F522A, D551R, I554A in A+B-K.O. Diploid strains containing plasmids with indicated genes fused to the GAL4 activation domain (AD) and GAL4 DNA binding domain (BD), were spotted as 10-fold serial dilutions on media lacking leucine, tryptophan, histidine, or adenine. 1mM 3-Amino-1,2,4-triazole (3AT) was added to decrease the background HIS3 expression (panels in 3rd. row). (**c**) RPA co-precipitates with PrimPol's RBD. Flp-In T-Rex-293 cells transfected with FLAG-tagged $\text{PrimPol}^{480\text{-}560}$ were grown in the presence or absence of doxycycline (10 ng/mL, 24 hrs), FLAG-$\text{PrimPol}^{480\text{-}560}$ was immunoprecipitated from the soluble cell lysate using anti-FLAG antibody and western blotted for PrimPol (anti-FLAG) and RPA (anti-RPA2). The presence and absence of doxycycline is indicated by +/- Dox, 'In' indicates the input, 'E1', 'E2', and 'E3', indicate elutions 1, 2, and 3, respectively. RPA was present in elutions 1 and 2 from cells grown in the presence of doxycycline, no RPA was observed in elutions from cells grown in the absence of doxycycline, thus confirming that WT FLAG-$\text{PrimPol}^{480\text{-}560}$ binds RPA *in vivo*.

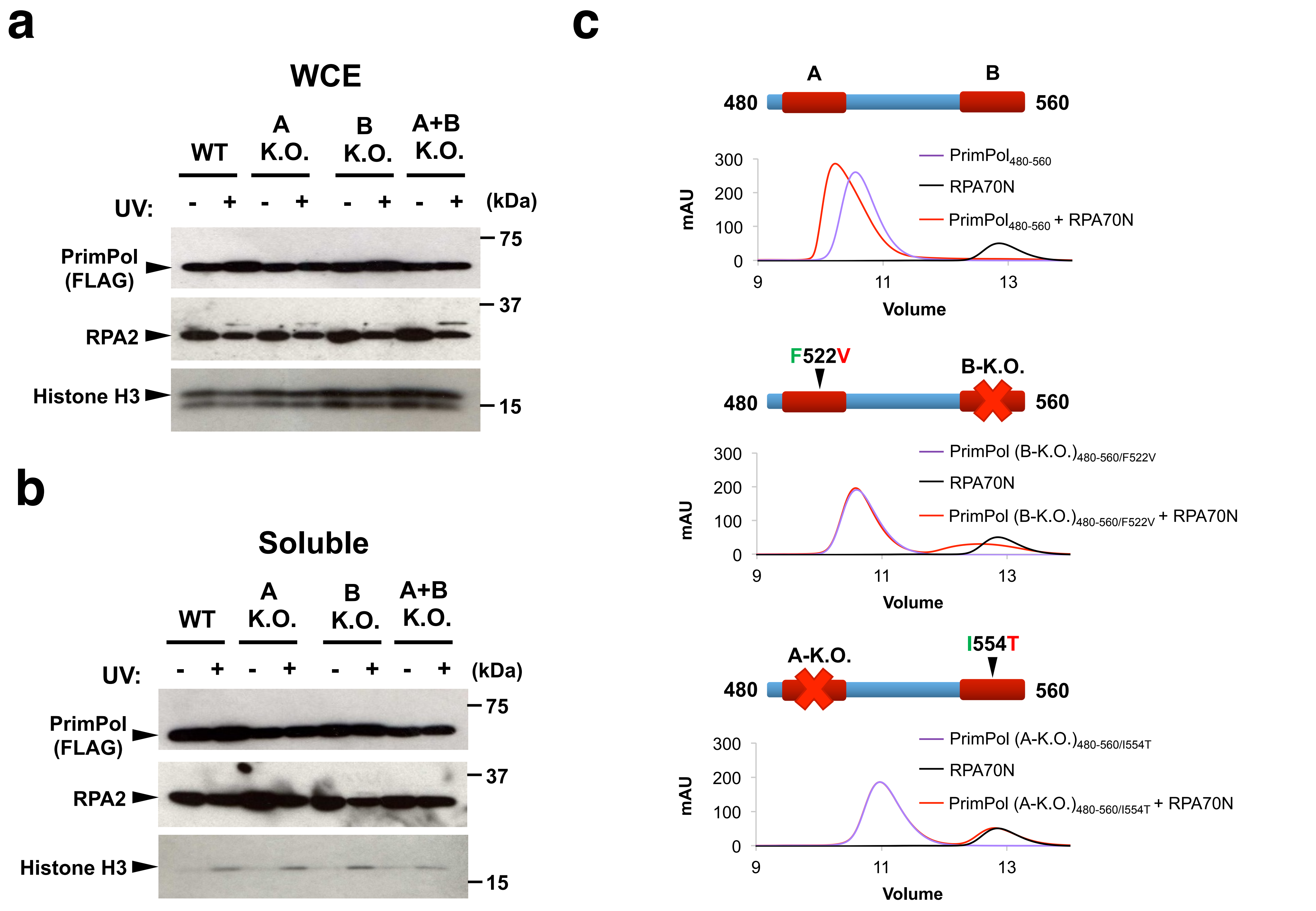

a
WCE
WT
A K.O.
B K.O.
A+B K.O.
UV: - + - + - + - +
(kDa)
PrimPol (FLAG)
RPA2
Histone H3
75
37
15
b
Soluble
WT
A K.O.
B K.O.
A+B K.O.
UV: - + - + - + - +
(kDa)
PrimPol (FLAG)
RPA2
Histone H3
75
37
15
c
A
B
480
560
PrimPol480-560
RPA70N
PrimPol480-560 + RPA70N
mAU
Volume
F522V
B-K.O.
PrimPol (B-K.O.)480-560/F522V
RPA70N
PrimPol (B-K.O.)480-560/F522V + RPA70N
A-K.O.
I554T
PrimPol (A-K.O.)480-560/I554T
RPA70N
PrimPol (A-K.O.)480-560/I554T + RPA70N

Supplementary Figure 6. Whole-cell extracts and soluble samples from PrimPol chromatin recruitment experiments. (**a**) Whole cell extract samples corresponding to the chromatin extraction experiment presented in Fig. 6a . (**b**) Soluble samples from the chromatin extraction experiment presented in Fig. 6a. (**c**) Analysis of the effect of PrimPol RBM mutations F522V and I554T, identified in cancer patient cell lines, on the RPA70N interaction. Chromatographs showing the retention volumes of unmodified $PrimPol_{480-560}$, $PrimPol_{480-560}$ F522V (on a B-K.O. construct), and $PrimPol_{480-560}$ I554T (on an A-K.O. construct), in the presence (red) and absence (purple) of RPA70N (black) in a 1:1 ratio. Unmodified $PrimPol_{480-560}$ is able to bind RPA70N (top panel). However, introduction of the mutations F522V (middle panel) or I554T (bottom) panel, in the absence of a second functional RBM, significantly abrogates binding to RPA70N.

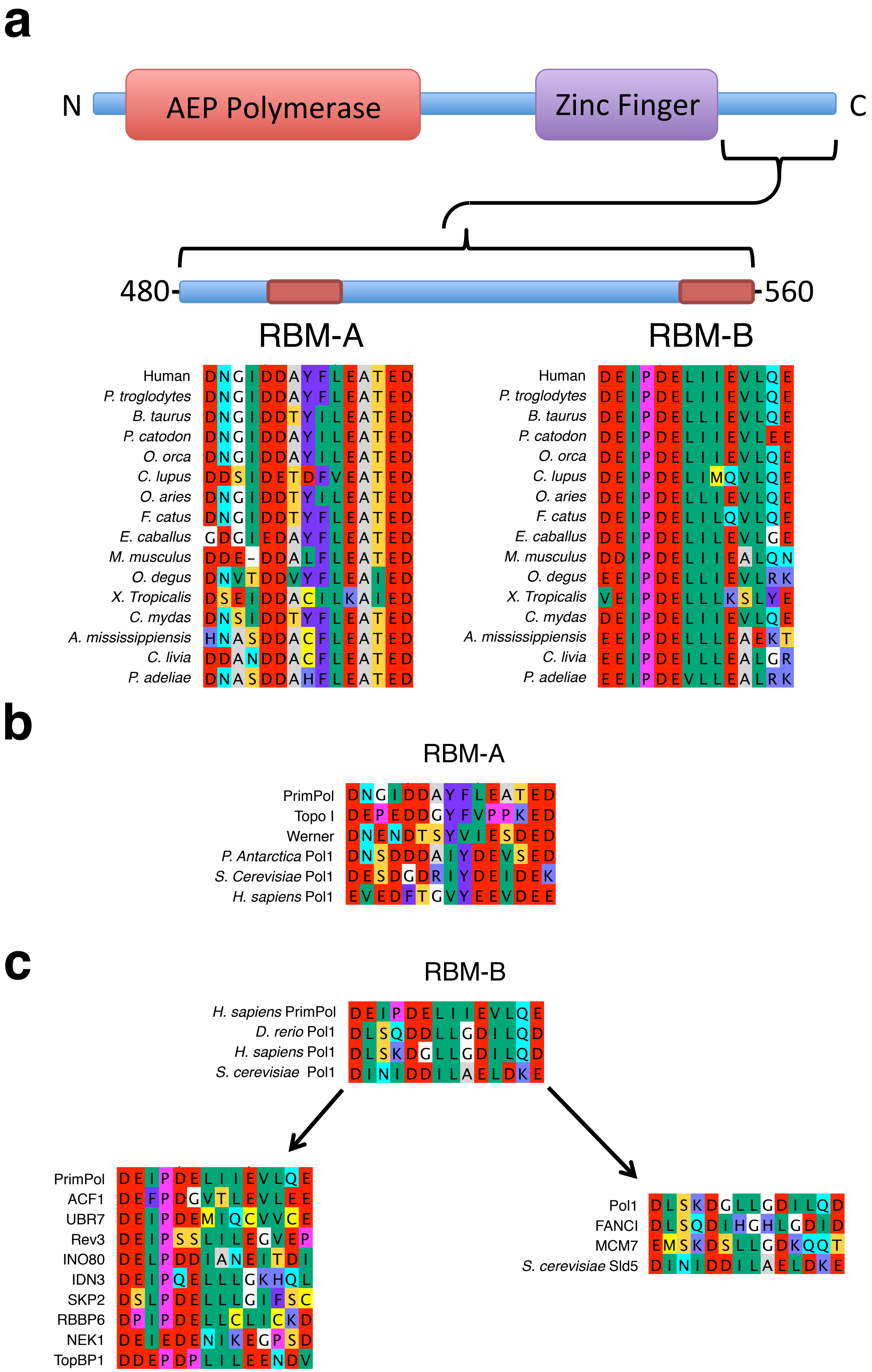

a
N
AEP Polymerase
Zinc Finger
C
480
560
RBM-A
Human DNGIDDAYFLEATED
P. troglodytes DNGIDDAYFLEATED
B. taurus DNGIDDTYILEATED
P. catodon DNGIDDAYILEATED
O. orca DNGIDDAYILEATED
C. lupus DDSIDETDFVEATED
O. aries DNGIDDTYILEATED
F. catus DNGIDDTYFLEATED
E. caballus GDGIEDAYFLEATED
M. musculus DDE–DDALFLEATED
O. degus DNVTDDVYFLEAIED
X. Tropicalis DSEIDDACILKAIED
C. mydas DNSIDDTYFLEATED
A. mississippiensis HNASDDACFLEATED
C. livia DDANDDACFLEATED
P. adeliae DNASDDAHFLEATED
RBM-B
Human DEIPDELIIEVLQE
P. troglodytes DEIPDELIIEVLQE
B. taurus DEIPDELLIEVLQE
P. catodon DEIPDELIIEVLEE
O. orca DEIPDELIIEVLQE
C. lupus DEIPDELIMQVLQE
O. aries DEIPDELLIEVLQE
F. catus DEIPDELILQVLQE
E. caballus DEIPDELILEVLGE
M. musculus DDIPDELIIEALQN
O. degus EEIPDELLIEVLRK
X. Tropicalis VEIPDELLLKSLYE
C. mydas DEIPDELIIEVLQE
A. mississippiensis EEIPDELLLEAEKT
C. livia EEIPDEILLEALGR
P. adeliae EEIPDEVLLEALRK
b
RBM-A
PrimPol DNGIDDAYFLEATED
Topo I DEPEDDGYFVPPKED
Werner DNENDTSYVIESDED
P. Antarctica Pol1 DNSDDDAIYDEVSED
S. Cerevisiae Pol1 DESDGDRIYDEIDEK
H. sapiens Pol1 EVEDFTGVYEEVDEE
c
RBM-B
H. sapiens PrimPol DEIPDELIIEVLQE
D. rerio Pol1 DLSQDDLLGDILQD
H. sapiens Pol1 DLSKDGLLGDILQD
S. cerevisiae Pol1 DINIDDILAELDKE
PrimPol DEIPDELIIEVLQE
ACF1 DEFPDGVTLEVLEE
UBR7 DEIPDEMIQCVVCE
Rev3 DEIPSSLILEGVEP
INO80 DELPDDIANEITDI
IDN3 DEIPQELLLGKHQL
SKP2 DSLPDELLLGIFSC
RBBP6 DPIPDELLCLICKD
NEK1 DEIEDENIKEGPSD
TopBP1 DDEPDPLILEENDV
Pol1 DLSKDGLLGDILQD
FANCI DLSQDIHGHLGDID
MCM7 EMSKDSLLGDKQQT
S. cerevisiae Sld5 DINIDDILAELDKE

Supplementary Figure 7. RBM-A and RBM-B represent common protein interaction motifs. (**a**) The domain architecture of PrimPol showing the positions of RBM-A and RBM-B relative to the functional domains of the protein. RBM-A and RBM-B motifs are conserved across chordate species. (**b**) Sequences similar to that of RBM-A are identified in Topoisomerase I and the Werner helicase. RBM-A of PrimPol aligns to a short acidic sequence in the N-terminus catalytic subunit of Pol α of *Pseudozyma antarctica*. A comparable sequence is found in the human homologue suggesting that this may be an RPA-interacting region of Pol α (Pol1). PrimPol RBM-B resembles the Ctf4-binding motif. The Ctf4-binding motif that has previously been identified in the catalytic polymerase subunit of Pol α and Sld5 of GINS is present in PrimPol at the C-terminus in RBM-B. This region of PrimPol interacts with RPA70N and represents a potential multiple protein binding motif. A PSI-BLAST of this motif from human PrimPol and Pol α identifies a number of other proteins involved in the metabolism of DNA.

a

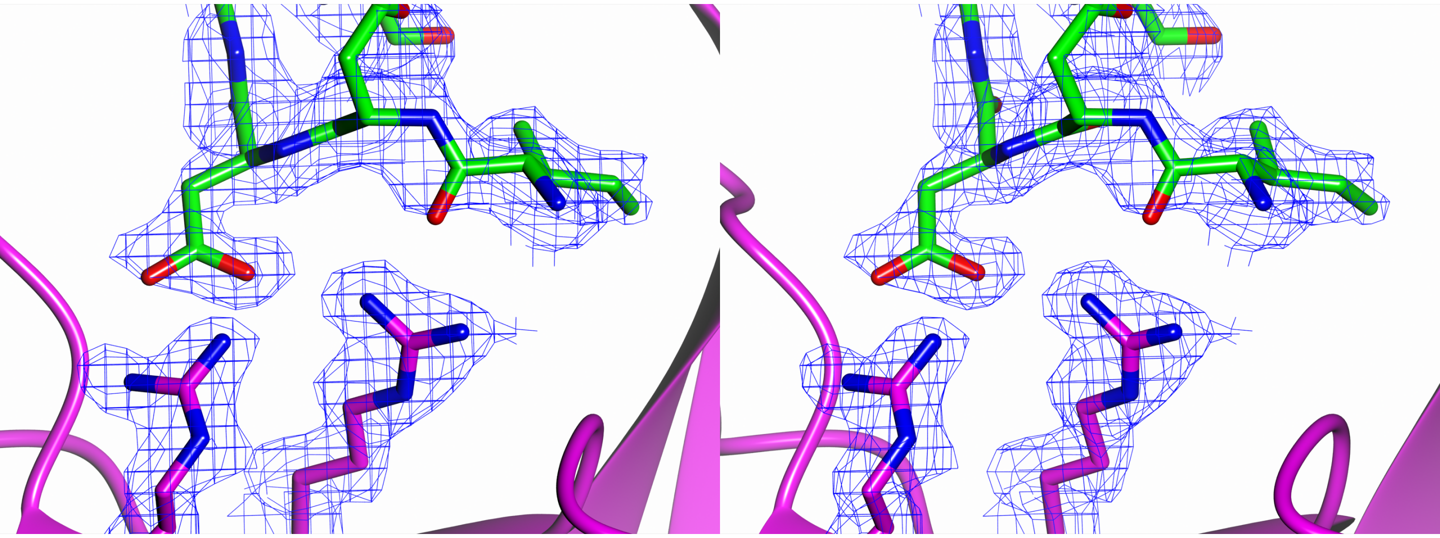

b

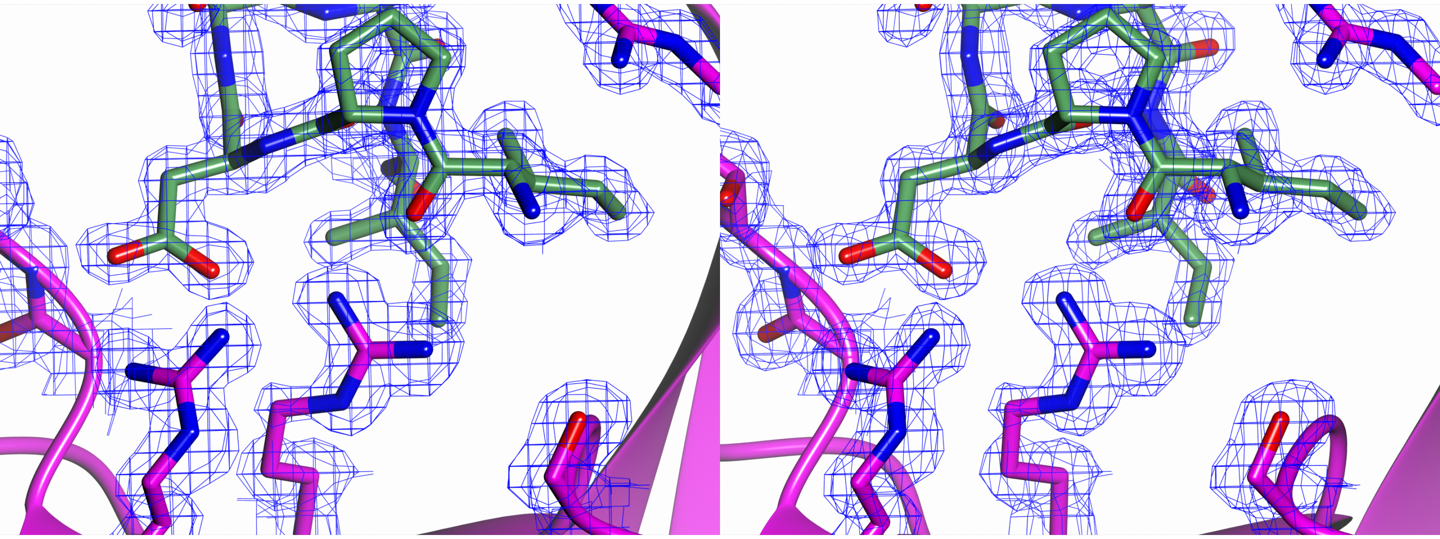

Supplementary Figure 8. Stereo views of electron density for RBM-A and RBM-B. (**a**) A stick representation of the residues I517-A520 of RBM-A (light green). Residues R43 and R31 from RPA70N that form the ionic interactions with RBM-A are also depicted (magenta). Density from a weighted 2Fo-Fc map scaled at 0.6σ is depicted in blue. (**b**) Residues I549-I554 of RBM-B are depicted (dark green). Residues R91, S54, R43, R31 and T34 from RPA70N involved in ionic interactions with RBM-B are also shown (purple). Density from a weighted 2Fo-Fc map scaled at 0.8σ is also depicted in blue.

Figure 4b

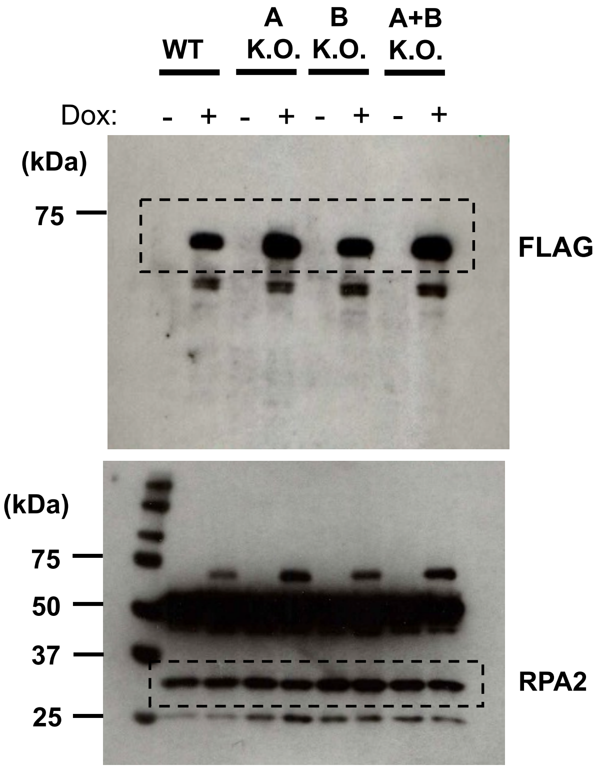

Figure 4c

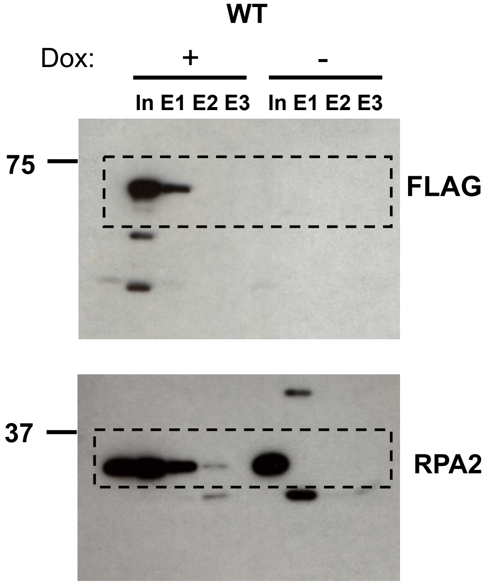

Figure 4d

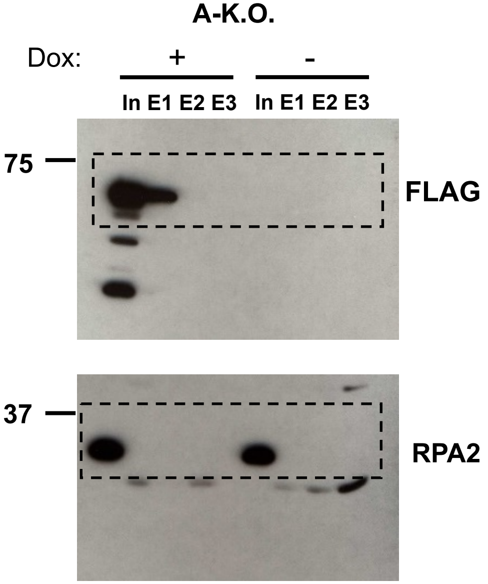

Figure 4e

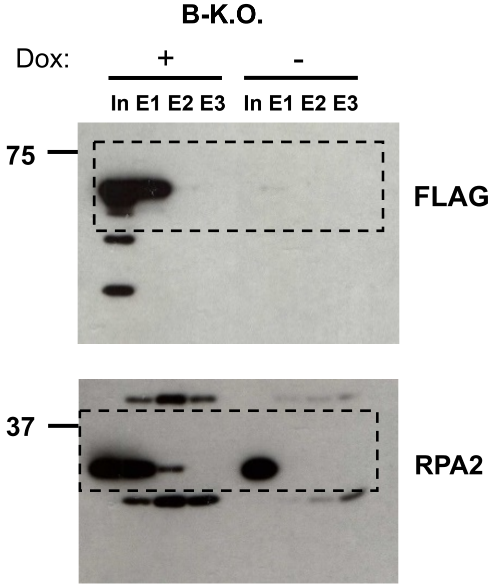

Figure 4f

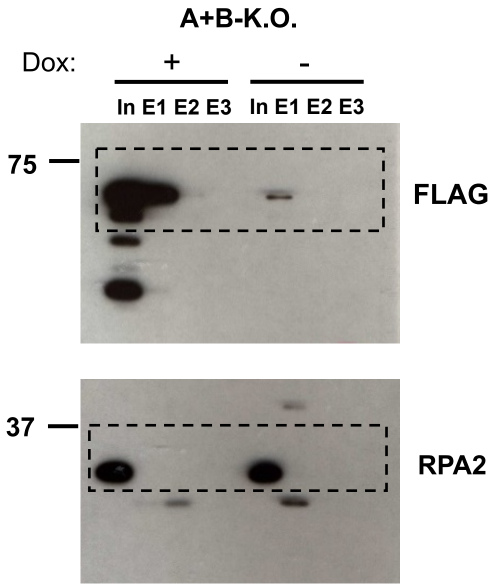

Figure 5a

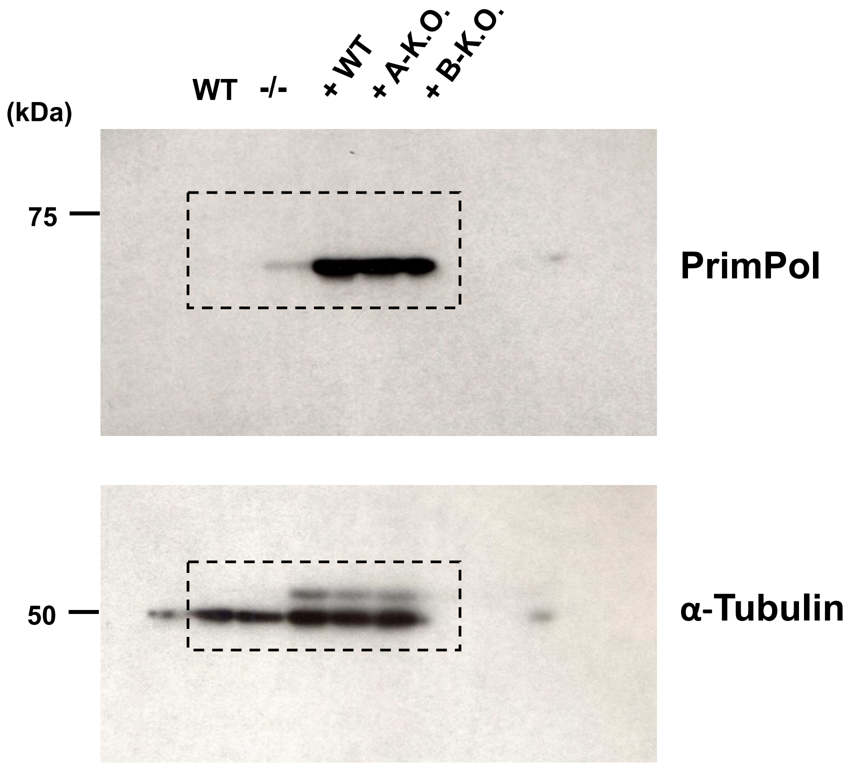

Supplementary Figure 5c

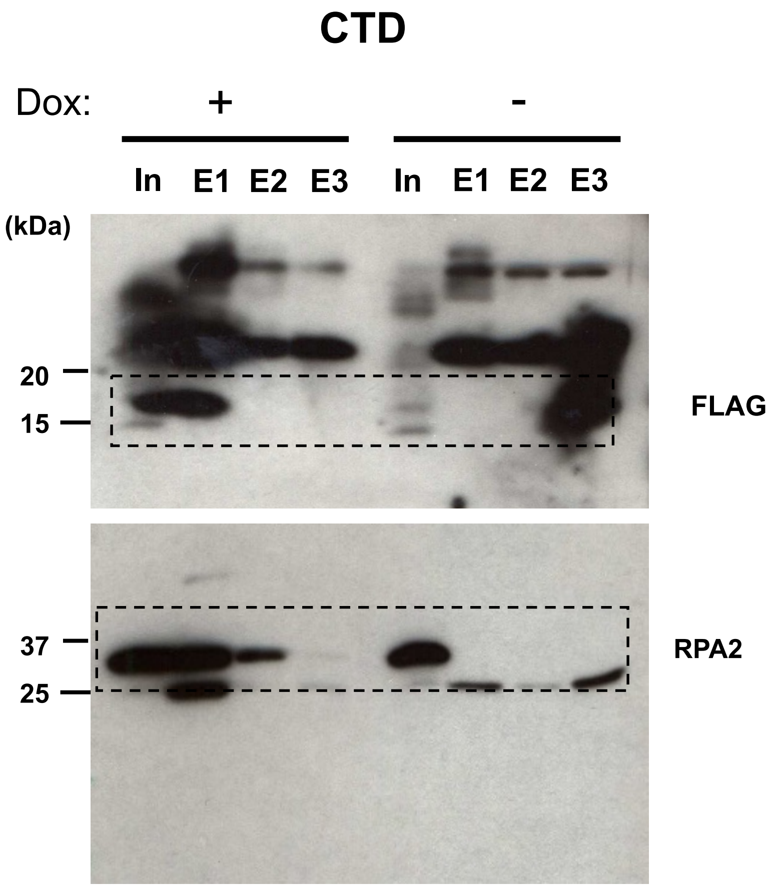

Figure 6a

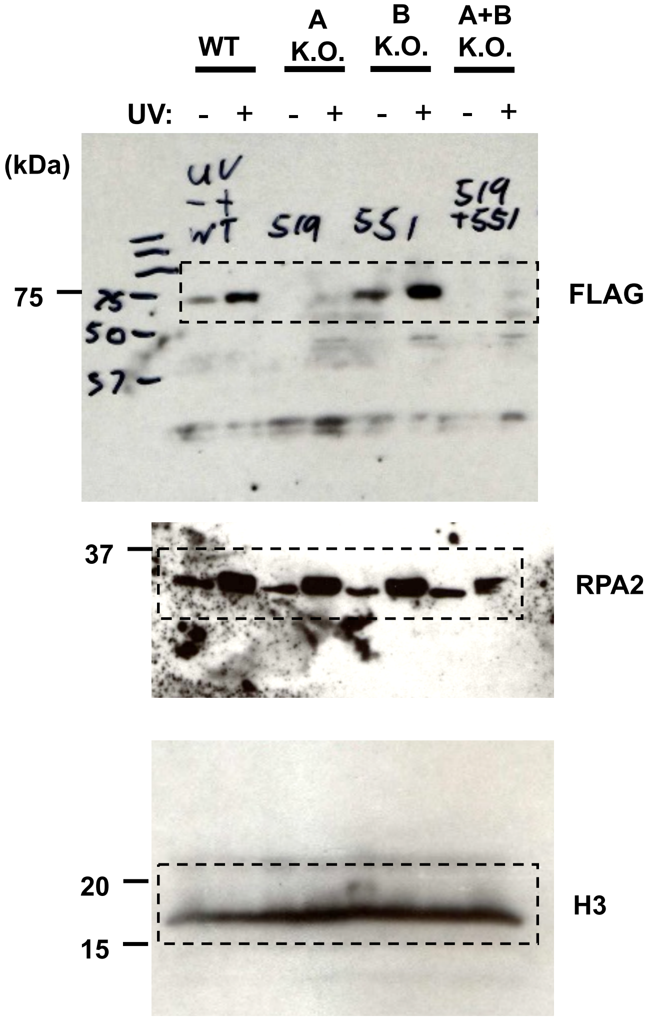

Figure 6b

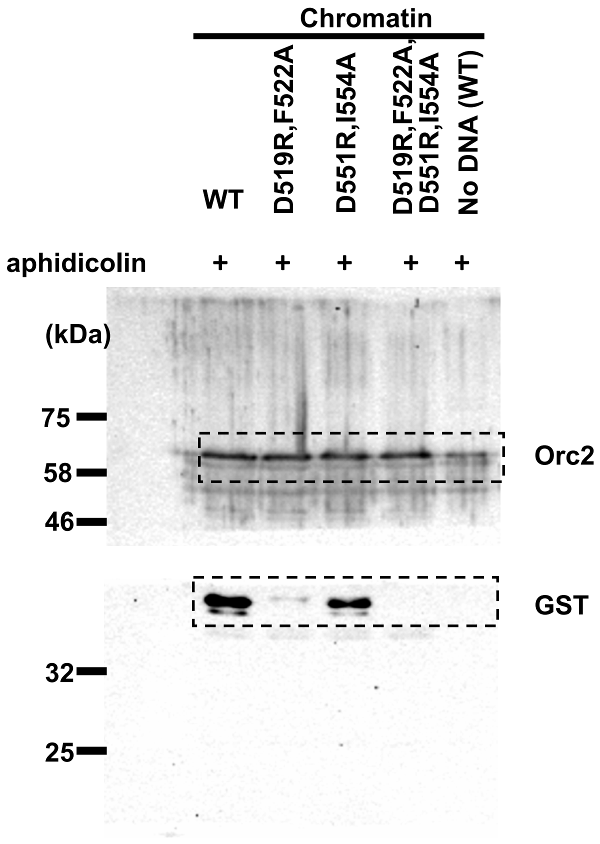

Figure 6c

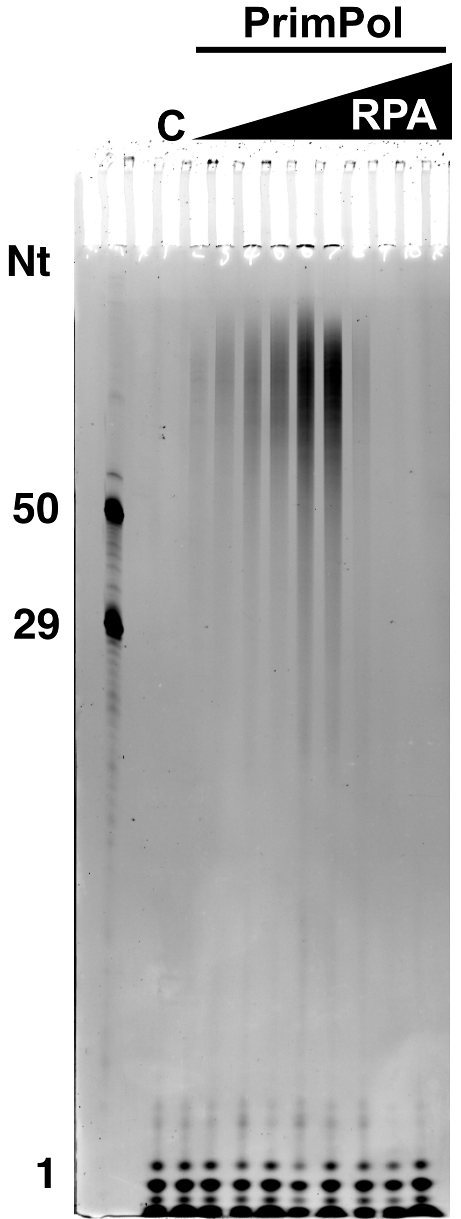

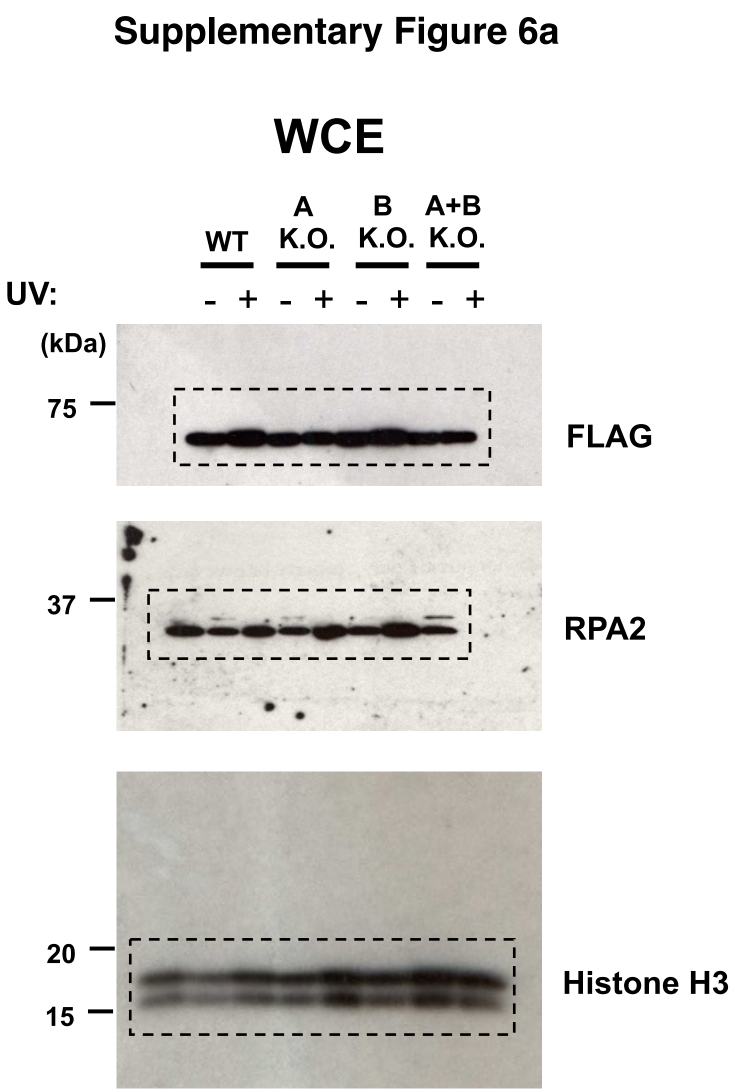

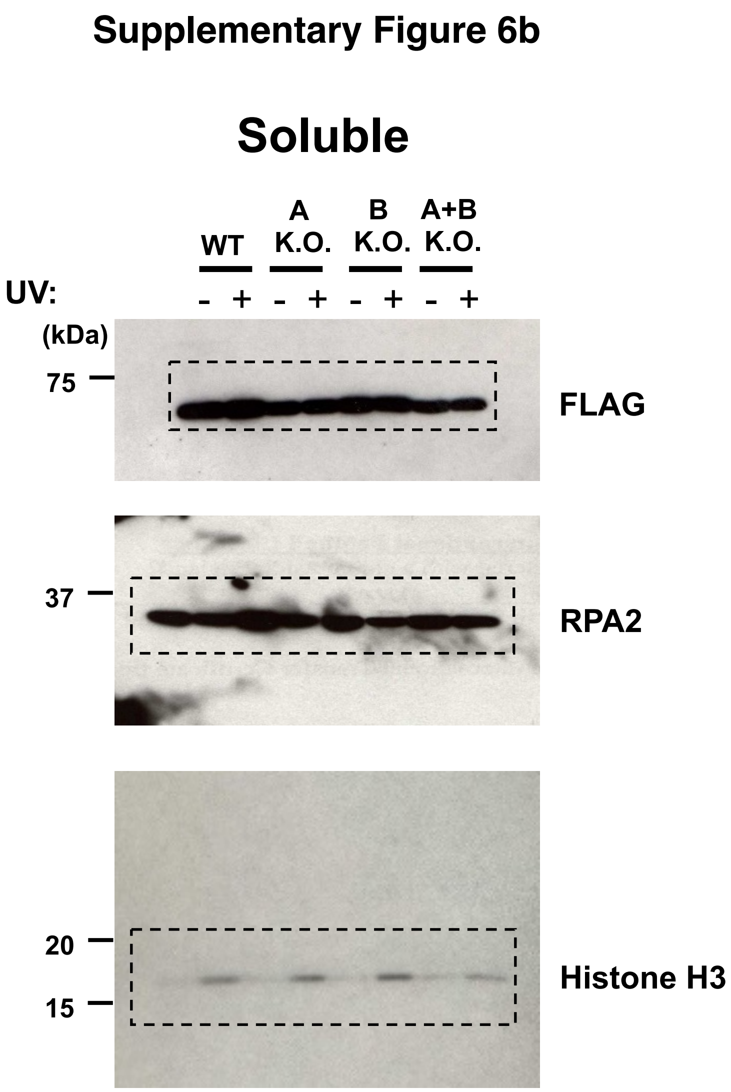

Supplementary Figure 9. Uncropped western blots and gels.

Supplementary Table 1

#	Oligonucleotide	Sequence
1	480 FWD	5′-GTTTCTTCATATGACAACAGATGAAGCAGATGAAAC-3′
2	560 REV	5′-CAAAGAAGCGGCCGCTTACTCTTGTAATACTTCTATAATTAGTTC-3′
3	1-546 FWD	5′- GTGAAGTGTAGTAAATTCCTGATGAACTAATTATAG-3′
4	1-546 REV	5′- CAGGAATTTACTACACTTCACTGTTATAACTGAG-3′
5	D514R/D518R/D519R FWD	5′-GGATCCGCCGCGCTTATTTTTTAGAAGCTACTGAAGATGCTGAATTAG-3′
6	D514R/D518R/D519R REV	5′-AAGCGCGGCGGATCCCATTTCTCCAGACAGCATCAGCAGATG-3′
7	D551R/I554A/I555A FWD	5′-TCTCAGTTATAACAGTGAAGTGGATGAAATTCCTCGCGAACTAGCGGCGGAAGTACTGCAGGAG-3′
8	D551R/I554A/I555A REV	5′-GGTGGTGGTGCTCGAGTGCGGCCGCTTACTCCTGCAGTACTTCCGCCGCTAGTTCGCGAGGAATTTC-3′
9	F522V FWD	5′-GATGATGCTTATGTTTTAGAAGCTACTGAAGATGCTGAATTAGCTGAAGC-3′
10	F522V REV	5′-CTTCTAAAACATAAGCATCATCAATGCCATTATCCCAGACAGCATC-3′
11	I554T FWD	5′-CTGATGAACTAACTATAGAAGTATTACAAGAGTAAGATCCGAATTCGAGCTC-3′
12	I554T REV	5′-ATACTTCTATAGTTAGTTCATCAGGAATTTCATCCACTTCACTGTTATAACTGAGAAG-3′
13	N-FLAG 1 FWD	5′-GTTTCTTGGATCCATGGATTACAAGGATGACGACGATAAGGGAAGCCATGGAAGCCATATGAATAGAAAA TGGGAAGCAAAACTG-3′
14	N-FLAG 480 FWD	5′-GTTTCTTGGATCCATGGATTACAAGGATGACGACGATAAGGGAAGCCATGGAAGCCATATGACAGATGAA GCAGATGAAAC-3′
15	D519R/F522A FWD	5′-GGCATTGATCGTGCTTATGCTTTAGAAGCTACTGAAGATGC-3′
16	D519R/F522A REV	5′-GCTTCTAAAGCATAAGCACGATCAATGCCATTATCCCAGAC-3′
17	D551R/I554A FWD	5′-GAAATTCCTCGTGAACTAGCTATAGAAGTATTACAAGAG-3′
18	D551R/I554A REV	5′-CTTCTATAGCTAGTTCACGAGGAATTTCATCCACTTCAC-3′

Supplementary Table 1: Sequences of the DNA oligonucleotide primers used for cloning and site-directed mutagenesis.
